# Why are human animacy judgments continuous rather than categorical? A computational modeling approach

**DOI:** 10.3389/fpsyg.2023.1145289

**Published:** 2023-06-05

**Authors:** Chris Westbury

**Affiliations:** Department of Psychology, University of Alberta, Edmonton, AB, Canada

**Keywords:** animacy, word embedding (word2vec), computational modeling methods, human judgment, taxonomy, classification

## Abstract

**Introduction:**

The concept of animacy is often taken as a basic natural concept, in part I because most cases seem unambiguous. Most entities either are or are not animate. However, human animacy judgments do not reflect this binary classification. They suggest that there are borderline cases, such as *virus*, *amoeba*, *fly*, and imaginary beings (*giant*, *dragon*, *god*). Moreover, human roles (*professor, mother, girlfriend*) are consistently recognized as animate by far less than 100% of human judges.

**Method:**

In this paper, I use computational modeling to identify features associated with human animacy judgments, modeling human animacy and living/non-living judgments using both bottom-up predictors (the principal components from a word embedding model) and top-down predictors (cosine distances from the names of animate categories).

**Results:**

The results suggest that human animacy judgments may be relying on information obtained from imperfect estimates of category membership that are reflected in the word embedding models. Models using cosine distance from category names mirror human judgments in distinguishing strongly between humans (estimated lower animacy by the measure) and other animals (estimated higher animacy by the measure).

**Discussion:**

These results are consistent with a family resemblance approach to the apparently categorical concept of animacy.

## 1. Introduction

The word animacy is defined in the Oxford English Dictionary (2022) as “The quality or condition of being alive or animate; animate existence; an instance of this.” This definition seems clear and unambiguous on its surface. However, when humans are asked to make judgments of animacy, they identify many intermediate or anomalous cases. The goal of the present paper is to use computational modeling to shed light on the lack of unanimous binary animacy decisions by English speakers for many words, by modeling the decisions for the 72 words rated for animacy in Radanović et al. (2016) and for 1,200 English words rated living/non-living from VanArsdall and Blunt (2022). I will consider two models with different set of predictors and synthesize their contributions to the understanding human animacy judgments at the end, by considering whether and why the models make the same kinds of errors that humans do.

As examples of the lack of agreement in animacy ratings, Radanović et al. (2016) reported that their university-student judges rated the animacy of giraffes or babies at about 50 (out of 100, where 0 = inanimate and 100 = animate), though we would normally think of the default state of these entities as living. This is approximately the same as the average ratings for balls (49.2) or snow (51.0), though we would not think of these entities as being alive. Other intermediate cases include imaginary beings such as ghosts (rated 41.7) and fairies (49.4); entities that imitate animate entities such as computers (52.2) and robots (33.1); and simple creatures such as amoebae (83.5) and viruses (69.4). Plants are a potentially ambiguous intermediate case, since they are animate by the Oxford English Dictionary’s definition, but we often interact with them in inanimate form. This may explain the lack of strong consensus about animacy in the ratings of words referring to plants such as *cabbage* (59.0), *tomato* (38.9), and *orchid* (59.0).

Languages that mark animacy grammatically can add additional complications within specific cultures. For example, in Cree, animal hides, trees (but not pieces of wood), and some (but not all) stones are marked grammatically as animate, perhaps (as suggested by Darnell and Vanek, 1976) reflecting that in Cree “a thing is classified as animate if it has power” (p. 164).

The role of animacy in semantic and lexical processing has been the focus of many studies (e.g., Cappa et al., 1998; Caramazza and Shelton, 1998; Grabowski et al., 1998; Mummery et al., 1998; Moore and Price, 1999; Tyler et al., 2000; Tyler and Moss, 2001; Radanović et al., 2016). Some studies have reported behavioral and/or neurological differences in response to animate and inanimate stimuli (Perani et al., 1995; Martin et al., 1996; Perani et al., 1999). Other studies have failed to replicate these findings (Devlin et al., 2002; Pilgrim et al., 2002; Tyler et al., 2003; Ilić et al., 2013). The linguistic encoding of animacy has been shown to affect many different aspects of psychological functioning, including the processing of relative clauses (Mak et al., 2002; Traxler et al., 2005; Gennari et al., 2012); attentional mechanisms (Bugaiska et al., 2019); the detection of semantic violations in language (Grewe et al., 2006; Szewczyk and Schriefers, 2011); the learning of artificial languages (Vihman et al., 2018); word recognition (Bonin et al., 2019) and the ability to recall words (Bonin et al., 2015; VanArsdall et al., 2015; Bugaiska et al., 2016; Popp and Serra, 2016, 2018; Nairne et al., 2017; Kazanas et al., 2020).

As Radanović et al. (2016) noted, one complication in studies using animacy is how stimuli are selected. Some studies have focused on only a few exemplars (i.e., tools versus animals, as in Perani et al., 1995, 1999; Martin et al., 1996). Others including a wider range of animate and inanimate stimuli.

Animacy ratings have been gathered in many languages (e.g., Serbian/English: Radanović et al., 2016; Portuguese: Félix et al., 2020; Persian: Mahjoubnavaz and Mokhtari, 2022; English: VanArsdall and Blunt, 2022). This study focuses on the two sets of English ratings in this list.

The first set was the set of 72 ratings from Radanović et al. (2016). As noted above, these were rated from 1 (inanimate) to 100 (animate). The authors reported that the English ratings were strongly correlated with independent Serbian ratings of the same words (*r* = 0.89, *p* < 0.001). They included a wide range of words. The ratings are summarized by into categories in Table 1. There is notable variation in ratings within categories of animate things. Contrary to the some claims (see discussion in Radanović et al., 2016, p. 17) human beings are rated as lower in animacy (Average [SD] rating: 60.0 [16.6]) than other animals (Average [SD] rating: 79.8 [22.6]; *t*(14.39) = 2.18; *p* = 0.046). Since human beings are certainly animate, this result is puzzling. I will consider it again in the conclusion section of this paper.

**Table 1:** Animacy ratings from Radanović et al. (2016), living/non-living ratings from VanArsdall and Blunt (2022), and model estimates of the latter, by category, ordered from most animate to least animate by human rating.

	Radanović et al.			VanArsdall and Blunt		Fitted
Category	Average	SD	*N*	Average	*N*	Model 2
Animal	79.8	22.6	9	96.2	96	89.2
Creature	76.1	18.3	8	94.0	33	90.1
Imaginary	64.2	18.5	7	49.1	18	62.7
Human	60.0	16.6	11	92.6	283	55.2
Plant	51.0	10.4	12	59.7	87	53.6
Natural	34.7	19.6	8	19.7	29	44.9
Artifact	33.0	22	17	17.1	273	50.0

The category ‘creature’ includes living beings other than mammals and birds, which are in the category ‘animal.’ The category ‘imaginary’ includes names for animate beings that do not actually exist, such as *unicorn* and *ghost*. The ‘natural’ category includes words referring to non-animate entities that occur naturally, such as *mud* and *salt*. The ‘artifact’ category includes words referring to non-animate entities constructed by human beings, such as *guitar* and *hat*. Radanović et al. are average animacy ratings, from 0 ‘Definitely inanimate’ to 100 ‘Definitely animate.’ The VanArsdall and Blunt (2022) living/non-living ratings have been converted from their original 7-point scale to be out of 100 as well. The final column includes fitted values from the living/non-living Model 2.

The other set is the recently released set of ratings from VanArsdall and Blunt (2022). They gathered living/non-living ratings from 1 to 7 for 1,200 English words. Each word was rated a minimum of 19 times (average [SD]: 25 [1.62]). The ratings are also summarized by category in Table 1.

These two sets of ratings are along slightly different dimensions. Some things that are clearly non-living (for example, unicorns and Santa Claus) might reasonably be judged animate. However, the larger set of ratings makes it possible to cross-validate the models, which is not possible with the small number of ratings from Radanović et al. (2016). Moreover, the ratings are correlated. The 50 words that appear in both data sets have animacy and living/non-living ratings that correlate at *r* = 0.60 (*p* < 0.0001).

The models use two different sources of data, to allow us to consider the issue from both a bottom-up perspective (to what degree is animacy encoded in semantics/patterns of language use?) and a top-down perspective (to what degree is animacy determined by membership in categories of animate entities?). One model uses the principal components of vector representations of words from a word embedding model (explained in more detail in the next section) to try to predict human ratings. This can give us an idea of the extent which animacy is encoded into language use, a bottom-up approach to animacy. The second model uses the similarity of a word’s vector to the vector of the names of definitely animate categories such as *human*, *animal*, and *plant*. This can give us an idea of the extent to which animacy is derivable from the goodness of its categorical membership. For example, though they are animals, humans are generally considered to be poor representatives of that class. It is possible that this is why humans are less likely to be judged as animate than other animals.

## 2. Model 1: introduction

The first model uses generalized additive models (GAMs) across the principal components (PCs) from a word-embedding model to predict human judgments. GAMs are able to capture non-linear relationships between predictors and a dependent measure but can also find linear relationships when they are the best fit for the data.

Word-embedding models are computational models that build vector representations of individual words that represent the average context in which that word appears in a large corpus of language. Perhaps the simplest way to do this is that used in the earliest model, Landauer and Dumais’s (1997) Latent Semantic Analysis (LSA), which built a *word x document matrix* in which the individual cells (prior to processing the matrix with singular value decomposition to reduce the dimensionality) recorded how often each word (rows) appeared in each document (columns). Since documents almost always have a semantic focus (they are usually *about* something), we might reasonably expect that words whose untransformed vectors were similar (say, vectors for the words *pet* and *cat*) are words that have similar semantics. Importantly, LSA does not directly measure whether *cat* and *pet* occur together in the same documents, which is what we call *first-order co-occurrence*. It measures whether the documents in which *cat* and *pet* appeared tended to contain the same words (a comparison of *word context* that we call *second-order co-occurrence*). It is possible for two words to have highly similar LSA vectors without ever appearing in the same document. For example, one can easily imagine that in some set of documents the informal word *cat* and the more formal word *feline* might never appear in the same document, but nevertheless would be likely to occur in documents that share many other words.

The basic principle of constructing vector representations of a word’s context continues in more recent word-embedding models, but the methods of constructing the vectors have been refined. There are two main differences. One difference is that most contemporary models do not construct their vectors from co-occurrence within *documents*, but rather from co-occurrence within some smaller moving window of text (which may be conceived of as very tiny documents, to keep the analogy with LSA precise). The second difference is that contemporary models do not merely count words but rather use more sophisticated computational methods to build the context vectors. In this paper I used a model called *word2vec* (Mikolov et al., 2013a,b,c). Skipping over some minor computational complications, word2vec models use a neural network with a single hidden layer (which is what is used as the vector representation of the word) to either predict the context of a target word (called *CBOW*, for *continuous bag of words*) or the inverse: to use context to try to predict a target word (*skipgram*, because the target word has been ‘skipped’ with context on either side). This paper uses the *skipgram* model with a 300-unit hidden layer and a context defined as two words on either side of the target word. Although these parameters are arbitrary, these values are commonly used in language research. For a corpus, I used the 150,000 most frequent words from a 100 billion words subset of the Google news corpus.^1^ To increase the chances that the results might have a clear interpretation, I applied principal components analysis (PCA) to this matrix, retaining all 300 principal components (PCs). The magnitude of the PC can thereby give us an estimate of how much variance in the matrix is accounted for.

Four words of the 1,200 words from VanArsdall and Blunt (2022) were eliminated from this study. The word *bluejay* was eliminated because it is confounded with the name of Canada’s favorite baseball team and appears only in capitalized form in the Google news corpus. The word is also problematic since the name of the bird is normally not considered a compound word but is rather composed of two words. Similarly, the word *hornet* appeared in the Google news corpus only in capitalized form (though it contained the plural form *hornets*), presumably referring to the name of the Marvel comic character. The word *ghoul* did not appear in the corpus, although *ghouls* did. The word *sphinx* did appear in the corpus, but only in capitalized form. The remaining dataset was randomly split into two sets of 598 words, with one half used for model development, and the other for cross-validation.

### 2.1. Model 1: method

All reported analyses were conducted in R 4.2.2 (R Core Team, 2022) using R Studio (2022.12.0 + 353; Posit Software, 2022) for macOS. The GAMs were analyzed using the mgcv package (v. 1.8–41, Wood, 2022; see also Wood, 2017).

Ninety-seven of the 300 PCs were significantly (*p* < = 0.05) correlated by GAM (i.e., possibly non-linearly) with the human animacy ratings from Radanović et al. (2016). This included 13 of the first 20 PCs (but not PC1). Since this provided more reliable predictors than there are data points, I used the 21 PCs that had a GAM whose output correlated with the human estimates at *p* < = 0.001 to construct a full GAM model. All predictors were entered initially. Those with the highest value of *p* were removed one by one until all remaining predictors entered with *p* < 0.05.

Ninety-two of the of the 300 PCs were significantly (*p* < = 0.05) correlated by GAM with the human living/non-living ratings from VanArsdall and Blunt (2022). This also provided more predictors than datapoints, because each smooth in the GAM has nine parameters using the default rank value (number of possible turning points, or *knots*) of 10 (the tenth is eliminated by centering the predictors). I therefore used the same method as above, initially entering only the 29 PCs that had a GAM whose output correlated with the human judgments at *p* < = 0.001. This included four of the first 20 PCs, but again, not PC1.

### 2.2. Model 1: result

**Figure 1:**
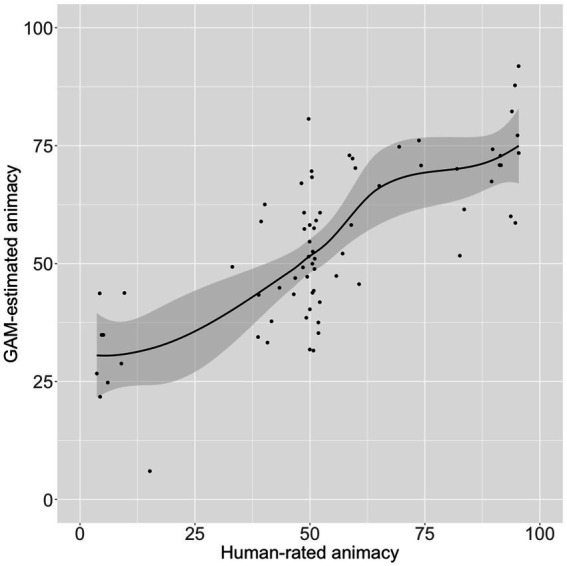
Human-rated (*X*-axis) versus GAM-estimated (*Y*-axis) animacy, using word2vec PCs as predictors. The gray-shaded area is the 95% confidence interval.

Only two PCs entered the model of the animacy ratings: PC123 and PC246. Together these PCs accounted for 56.6% of the variance in those ratings (*p* < 0.00001; see Figure 1).

I constructed a dictionary by taking the 75,000 most frequent words from Shaoul and Westbury, 2006. I eliminated words that did not appear in the Google news matrix, which does not include closed class words, as well as compounds words (or phrases) with spaces in them. The final dictionary contains 67,717 words. Applying the GAM to this dictionary suggested that the model may be over-fit to the small data set, since the words estimated most highly animate were not clearly exemplars of any animate category. The top 10 words were *disclaims, threes, clientless, fouling, republication, desegregation, effigies, barriers, reflate*, and *mineralization*.

Four PCs entered the living/non-living model: PC30, PC138, PC248, and PC225 (see Table 2). Together these PCs accounted for 12.5% of the variance in the human ratings (*p* < 0.00001). The model did not cross-validate successfully. Its predictions were unreliably correlated (*r* = −0.02, *p* = 0.61) with the human living/non-living ratings in the validation set.

**Table 2:** Best GAM model to predict the living/non-living ratings from VanArsdall and Blunt (2022), using word-embedding PCs as predictors.

PREDICTOR	RANK	df	*F*	*p*
PC30	5.00	6.09	3.25	0.004
PC138	6.28	7.46	2.30	0.020
PC225	8.43	8.91	3.12	0.001
PC248	3.06	3.93	2.78	0.026

### 2.3. Model 1: discussion

Although the models did not generalize well to the full dictionary or to a validation dataset, we can draw some tentative conclusions from this initial model.

The lack of good generalization and the lack of concordance between the two models suggests that one conclusion we can draw is that little of the variance in animacy or living/non-living judgments can be derived from the PCs in a word embedding model. The failure of these ‘bottom-up’ models suggests that animacy or being alive are not strongly encoded in patterns of word use. More speculatively, we can conclude that animacy is not a basic component of lexical semantics, since many components considered to be basic can be well-estimated from the PCs (e.g., see Hollis and Westbury, 2016; Hollis et al., 2017; Westbury and Hollis, 2019).

However, that said, the second conclusion is that animacy may be correlated with other aspects of semantics, since a large number of individual PC GAMs produced estimates that were reliably correlated with the human animacy ratings. The Radanović et al. (2016) are reliably correlated with the extrapolated estimates of human judgments of valence, dominance, and arousal from Hollis et al. (2017). Higher animacy ratings are associated with lower valence (*r* = −0.29, *p* = 0.01), higher arousal (*r* = 0.35, *p* = 0.003), and lower dominance (*r* = −0.33, *p* = 0.005). The negative correlation with dominance reflects the fact that many small (i.e., low dominance) living things such as *amoeba* (83/100), *bacteria* (83/100), *squirrel* (95/100), *worm* (92/100), and *spider* (94/100) receive high animacy ratings. The animacy ratings are also positively correlated (*r* = 0.31, *p* = 0.007) with the measure of self-relevance (how strongly a word is associated with the first-person singular word *I*) that was defined in Westbury and Wurm (2022), where it was shown to strong predictor of the value of early PC values across a large dictionary. A GAM developed with all these values to predict the animacy ratings allowed in only arousal and self-relevance with *p* < 0.05. Together these two measures accounted for 21.2% of the variance in the ratings.

A third conclusion is that (tautologically) most of the error in predicting animacy is seen for words of ambiguous animacy. In Figure 1 there is a wide range of model estimates for words that were rated the mid-range of animacy by humans in Radanović et al. (2016).

## 3. Model 2: introduction

Word2vec vectors for category names (such as the vector representing the word *animal*) usually (though not necessarily) serve as centroids for the category they name. This means that words with vectors that are similar (by cosine distance) to the vector for a category name are often members of that category. For example, the twenty vectors most similar to the vector of the word *vegetable* are the vectors for the words *tomato, potato, tomatoes, broccoli, sweet_potato, onion, onions, cauliflower, mango* (oops!), *kale, potatoes, mangos, cabbage*, and *melons*. We may perhaps forgive the model for sometimes confusing vegetables and fruit, since we ourselves routinely refer to the tomato fruit, the avocado fruit, the olive fruit, the cucumber fruit, the zucchini fruit, and several other fruits (strictly speaking, plants in which the edible part develops from a flower) as vegetables. If humans discuss fruits as if they were vegetables, we must expect that word embedding models will reflect that. Of course, fruits and vegetables also do correctly both belong to many other categories: *plant products, things we eat, things that cannot thrive in freezing weather, domesticated products, things you will find at the grocery store*, *everyday objects, things that can be composted*, and so on. A word-embedding model of categorization may be influenced by all these categories simultaneously since it can only induce the categories from the similarity of the contexts of words as encoded in the words’ vectors. It is possible that a super-ordinate category could be better captured by patterns of word use than a more focal category, if people used language in a way that better reflected that super-ordinate category.

In the second model, this categorizing feature of word embedding models is used, by building models of the human ratings based on the distance from the vectors of the names of categories of unambiguously animate things.

### 3.1. Model 2: method

For predictors I used the cosine similarity of each word that had been classified by humans to five main category names of definitely animate and living things: *plant, animal, insect, human,* and *bacteria*. Of the five taxonomic kingdoms, three are captured by these categories (plant, animal, and bacteria/~monera). The other two (funghi and protista) are less relevant kingdoms when it comes to animacy. Insects and humans are broken out of the animalia kingdom to which they belong because they are regularly incorrectly classified as non-animate.

### 3.2. Model 2: results

**Figure 2:**
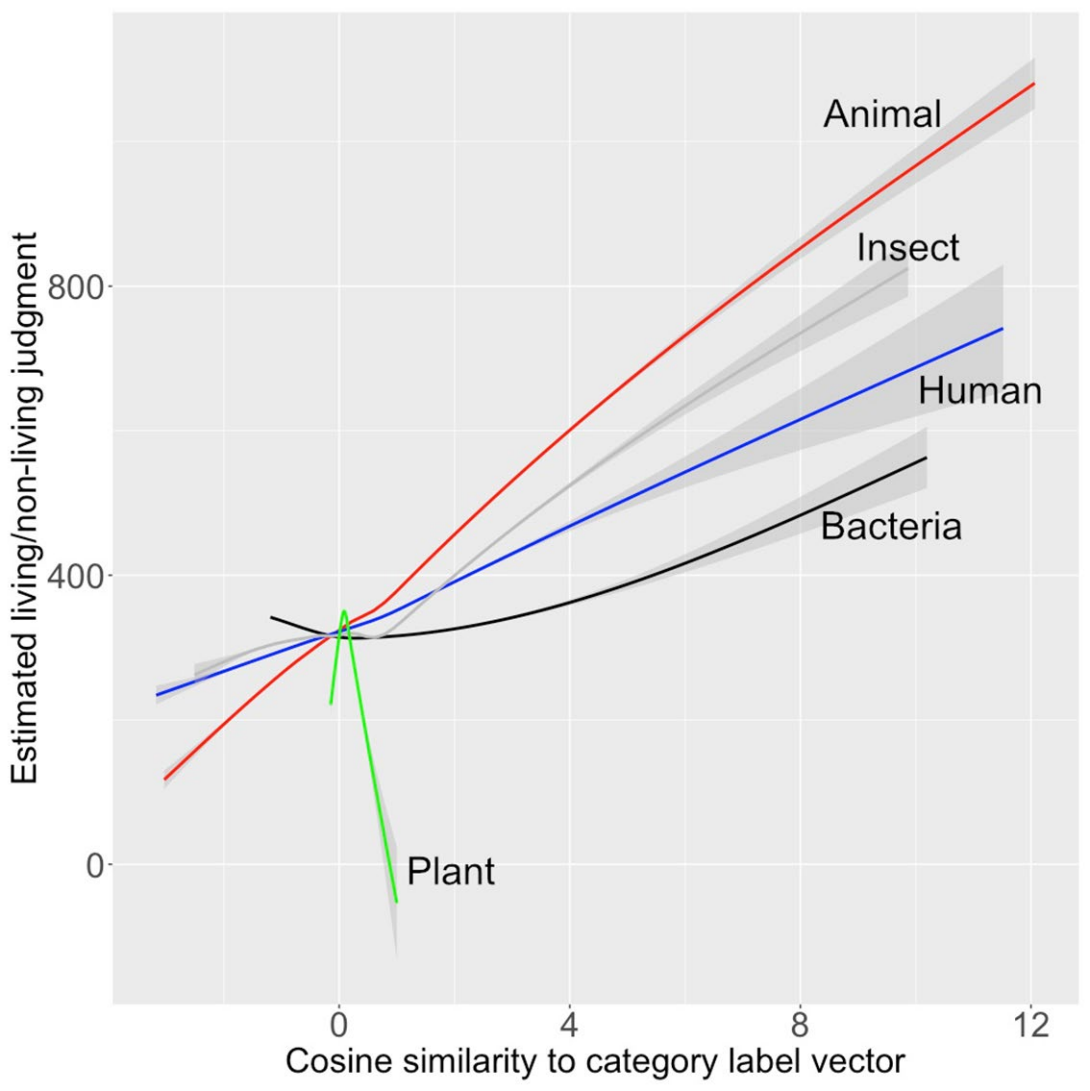
Pearson correlations between category-name predictors and human ratings.

The Pearson correlations between all the predictors and the ratings from Radanović et al. (2016) are shown in Figure 2. The correlations between the human ratings for each word and the cosine distance of their word2vec vectors from the vectors of the category labels were reliable at *p* < 0.001 for all categories except *plant* (*r* = 0.13, *p* > 0.05).

**Figure 3:**
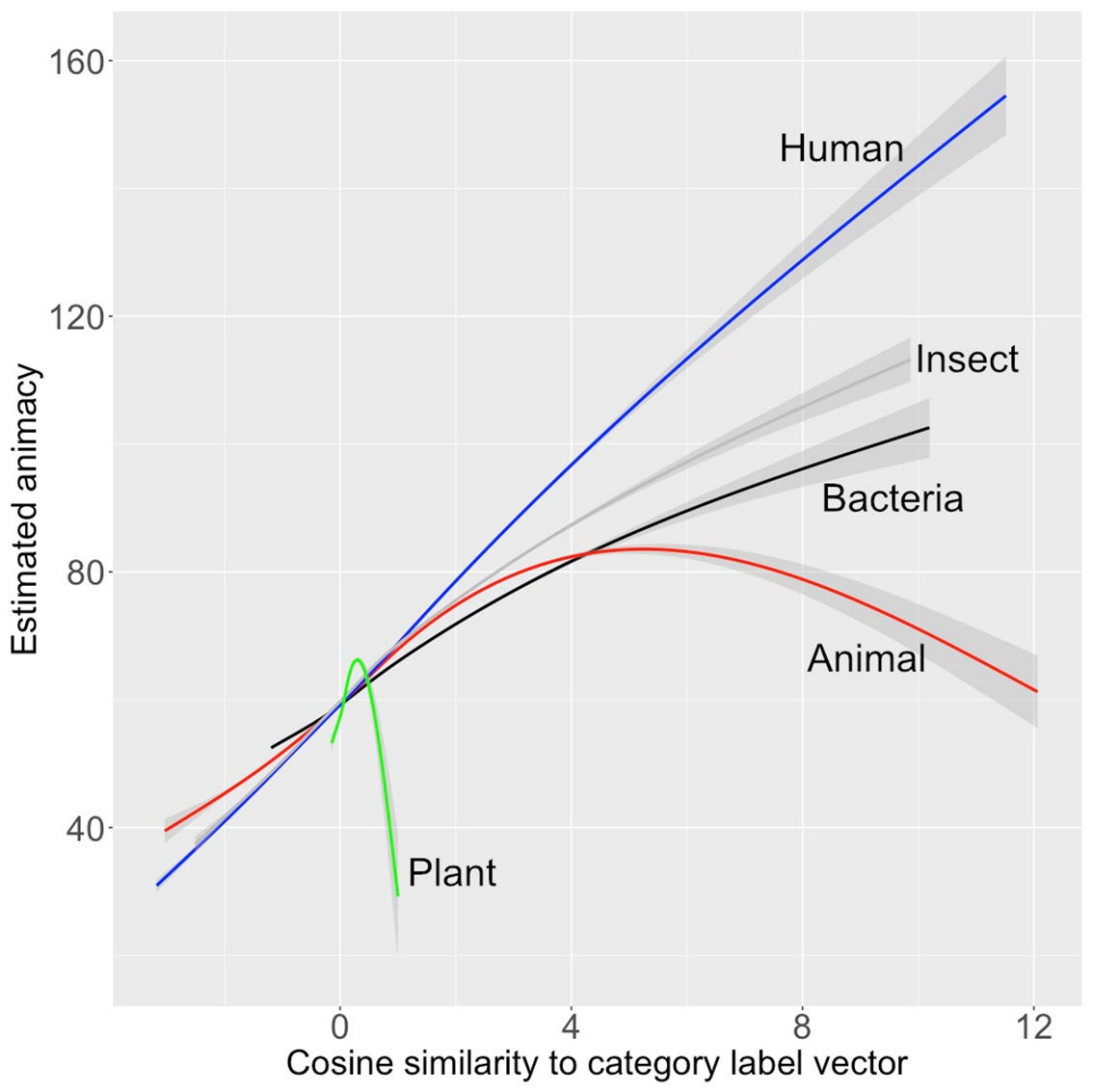
Model 2’s estimates of animacy (*y*-axis) graphed against the normalized cosine similarity of the vectors for the category labels of 67,717 words (*x*-axis) The gray-shaded area is the 95% confidence interval.

The best GAM model to predict the Radanović et al. (2016) ratings included only two predictors that entered with *p* < 0.05, *insect* and *human*. This model is summarized in Table 3. It accounted for 28.8% of the variance in the human ratings. The relationship between the predictors and the model estimates for are shown graphically in Figure 3.

**Table 3:** Best GAM model to predict the animacy ratings from Radanović et al. (2016), using cosine distance to category label vectors as predictors.

Predictor	Rank	DF	*F*	*p*
Insect	1.00	1.00	9.64	0.003
Human	1.00	1.00	7.24	0.009

When the model was applied to the full dictionary, the 10 words estimated most animate were almost all insects: *beetle, aphid, beetles, moth, pests, pest, aphids, wasps, wasp,* and *fungus*.

**Figure 4:**
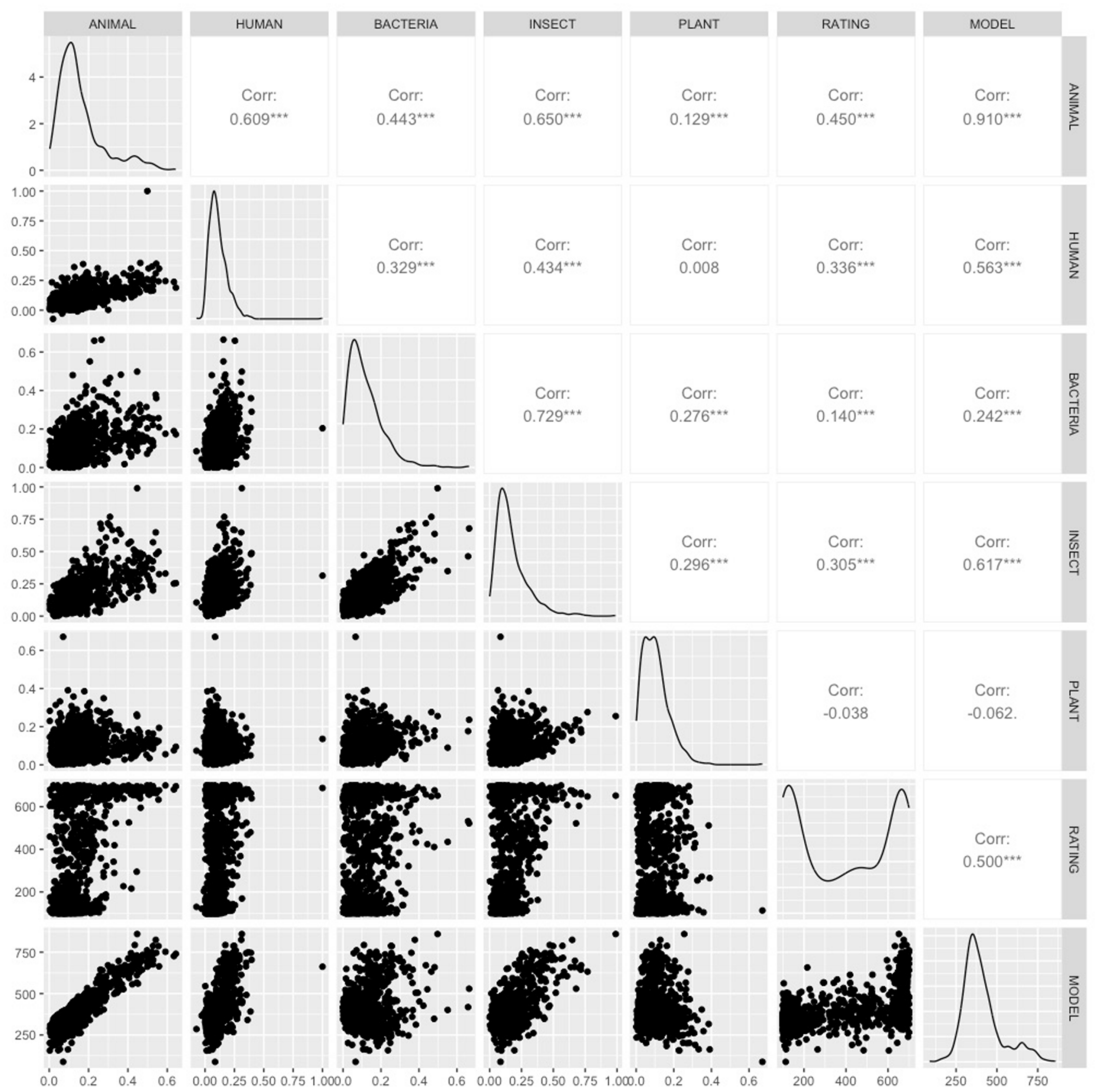
Relationships between human living/non-living ratings, Model 2’s estimates of those ratings, and vector cosine similarity of each word’s vector to five animate category labels.

The living/non-living judgments from VanArsdall and Blunt (2022) were modeled in the same way. Four predictors entered with *p* < 0.05: *animal, bacteria, insect* and *plant* (see Table 4). The model accounted for 26.7% of the variance in the human ratings in the development set and 23.7% of the variance in the validation set. The model was applied to the full dictionary. The 10 words estimated most animate were *animal, insect, rodent, animals, owl, bird, reptile, critter, feline,* and *elephant*. This list has high face validity, both because it only includes only words that name living things and because it includes many high-level living-thing category names. The relationship between the predictors and the living/nonliving judgments are shown graphically in Figure 4.

The predictions from this model are broken down into categories in the rightmost column of Table 1. The seven average categorical predictions from the model are highly correlated with the average categorical human ratings of both animacy (*r* = 0.91, *p* = 0.002 one-sided) and living/non-living (*r* = 0.81, *p* = 0.01 one-sided) (Table 4).

**Table 4:** Best GAM model to predict the living/non-living ratings from VanArsdall and Blunt (2022).

Predictor	Rank	DF	*F*	*p*
Animal	1.91	2.40	25.79	<2e-16
Bacteria	1.67	2.10	5.57	0.004
Insect	2.87	3.62	3.10	0.020
Plant	3.34	4.13	2.54	0.041

Using cosine distance to category label vectors as predictors.

### 3.3. Model 2: discussion

The results from all models to predict human animacy and living/non-living ratings are summarized in Table 5. There are two main findings.

**Table 5:** Results for predicting human animacy ratings, using GAM with PCs (Model 1) or cosine similarity to the vectors for label names (Model 2).

Predictors	Data	*R*^2^
PCs	Animacy	0.566
PCs	Living [Dev]	0.125
PCs	Living [Val]	0
Cosine	Animacy	0.288
Cosine	Living [Dev]	0.267
Cosine	Living [Val]	0.237

One is that modeling human animacy and living/non-living judgments using distance from category names is more successful than modeling them using word2vec PCs. Although the word2vec PCs predicted the 72 animacy judgments relatively well (*R*^2^ = 0.57), that model had very low face validity when extended to the whole dictionary. Those word2vec vectors were also poor at predicting the living/non-living judgments. The best model accounted for only 12.5% of the variance and failed to cross-validate at all. In contrast, the model using distances from category names accounted for roughly the same variance in the animacy (28.8%) and living judgments (26.7%), although of course there are many more living judgments. That model cross-validated relatively well, accounting for 23.7% of the variance in the living/non-living judgment validation dataset. It also had high face validity when applied to a larger set of words.

The other finding of interest is that neither of models using categorical distance included distance from the category *human*. This is noteworthy because (as shown in Table 1 and discussed above) human categories tend to be rated low by humans on both animacy and (to a lesser extent) living/non-living judgments, where they received an average rating of 92.6/100, compared to 94.0/100 for mammals and birds and 94.0/100 for other living creatures.

## 4. General discussion

Of course, if we provided a model of animacy with categorical information, it would achieve perfect classification, since the five categories of plant, animal, insects, humans (which are of course also animals, but we generally do not speak of them this way), and bacteria cover the superordinate category of the animate almost perfectly. The fact that humans are not unanimous about their decisions suggests that human beings must not be relying on categorical information, which we already knew from their failure to accept members of these categories as animate with perfect accuracy.

The fact that the pattern of errors in the models is similar to the pattern of errors seen in humans suggests that human may be making animacy decisions based on contextual information (or the categories that may be derived from that information) rather than on category membership.

The model which used cosine distance from category labels performed much better at classifying words as being animate than the analogous model that used PCs. We can roughly conceive of the models as being bottom-up (PC predictors) versus top-down (category label predictors). These results therefore suggest that animacy is unlike valence or arousal, which are usually conceived as being components of semantics (Osgood et al., 1957). It is rather more like *being expensive* or *being soft*, an objectively grounded top-down classification that we learn from experience.

The second noteworthy finding supports this. That is the fact that the models built on cosine distance from the category name vectors make one of the same errors that humans do: they tend to rate humans as lower in animacy than animals. Table 1 shows that human beings rated human words (such as *mother, boy,* and *professor*) as animate at 60/100, compared to 79.8 for animal names. Similarly, the model rates humans at 55.2, compared to 89.2 for animals. This may reflect that humans are not generally conceived of (or, at least, written about) as animate.

The model also replicates humans in (erroneously) classifying plants as moderately animate. Humans rated plants at 51/100 (Table 1). The model rates them at 53.6/100.

The top 200 most animate words according to the final model are reproduced in Supplementary Appendix 1. Animacy ratings for the full dictionary of 67,717 words are available at https://osf.io/k3cn9/.

It is obvious that humans do not make animacy decisions using category membership. If they did their animacy ratings would be unanimously high or low for many words that get intermediate ratings. The success of the category vector distance models at modeling human ratings suggests that humans are instead making animacy judgments by making rough animate category membership judgments (without considering the category of humans, according the best model discussed above). This may have implications for studies looking at animacy effects. The repeated finding that humans and living things outside of the animalia kingdom are poorly classified as animate by models using cosine distance from the vectors of category names suggests that language use does not present humans and living things outside of the animalia kingdom in contexts that highlight their animacy. These results suggest that humans make animacy ratings not by considering the category of each item, but rather by making family resemblance judgments to animate categories. The nature and direction of those judgments are reflected in word-embedding models.

## Data availability statement

The original contributions presented in the study are included in the article/Supplementary material, further inquiries can be directed to the corresponding author.

## Ethics statement

The studies involving human participants were reviewed and approved by the University of Alberta Research Ethics Board. The patients/participants provided their written informed consent to participate in this study.

## Author contributions

The author confirms being the sole contributor of this work and has approved it for publication.

## Funding

This work was funded by the Natural Sciences and Engineering Research Council of Canada, grant#RGPIN-2018-04679.

## Conflict of interest

The author declares that the research was conducted in the absence of any commercial or financial relationships that could be construed as a potential conflict of interest.

## Publisher’s note

All claims expressed in this article are solely those of the authors and do not necessarily represent those of their affiliated organizations, or those of the publisher, the editors and the reviewers. Any product that may be evaluated in this article, or claim that may be made by its manufacturer, is not guaranteed or endorsed by the publisher.

## Footnotes

^1^ https://code.google.com/archive/p/word2vec/
