# Supplementary material for: Why are human animacy judgments continuous rather than categorical? A computational modeling approach
Source: Front Psychol. 2023 Jun 5;14:1145289. doi: 10.3389/fpsyg.2023.1145289 (PMC10278539; doi:10.3389/fpsyg.2023.1145289)
Supplement: Supplementary file 1 [file Data_Sheet_1.docx]

Appendix A: 200 words estimated highest on living/non-living, by using a GAM over cosine similarity to the vectors for the words *animal, insect, human,* and *bacteria.*

| animal |
| --- |
| insect |
| rodent |
| animals |
| owl |
| bird |
| reptile |
| critter |
| feline |
| elephant |
| raptor |
| orangutan |
| cat |
| primate |
| coyote |
| wildlife |
| mammal |
| alligator |
| ape |
| avian |
| dog |
| cougar |
| rabbit |
| reptiles |
| amphibian |
| snake |
| panther |
| gorilla |
| coati |
| wolf |
| arthropod |
| carnivore |
| pet |
| llama |
| ungulate |
| critters |
| lizard |
| pachyderm |
| tiger |
| panda |
| iguana |
| tortoise |
| rodents |
| felines |
| toad |
| housefly |
| cats |
| insects |
| elephants |
| pigeon |
| possum |
| goshawk |
| chimp |
| bobcat |
| herpetological |
| canine |
| frog |
| lemur |
| emu |
| creatures |
| squirrel |
| rabbits |
| monkey |
| goat |
| zoological |
| deer |
| creature |
| starling |
| wallaby |
| otter |
| rhino |
| primates |
| coatis |
| mandrill |
| ant |
| fox |
| cheetah |
| raptors |
| avians |
| basset |
| equine |
| lizards |
| cottontail |
| piglet |
| turtle |
| falcon |
| cavy |
| koala |
| raccoon |
| rattlesnake |
| greyhound |
| mantids |
| cassowary |
| coyotes |
| anteater |
| peregrine |
| pug |
| puma |
| armadillo |
| birds |
| chimpanzee |
| bruin |
| tigers |
| gibbon |
| jaguar |
| jackrabbit |
| liger |
| lyrebird |
| beagle |
| foxes |
| kitten |
| boar |
| chimpanzees |
| tarantulas |
| grizzly |
| cavies |
| arachnid |
| gyrfalcon |
| ornithological |
| mandrills |
| ringtails |
| cockroach |
| owlet |
| mustang |
| kinkajou |
| puppy |
| elk |
| cockatoo |
| arboreal |
| livestock |
| snakes |
| giraffe |
| guanacos |
| peafowl |
| furry |
| feral |
| simians |
| purebred |
| dogs |
| possums |
| herpetology |
| gator |
| undomesticated |
| bear |
| porcupine |
| rhinoceros |
| wasp |
| pachyderms |
| hornbill |
| locust |
| badger |
| peahen |
| shrike |
| wolverine |
| bisons |
| antelope |
| owls |
| beaver |
| foxhound |
| tigress |
| ostrich |
| ptarmigans |
| kinkajous |
| pig |
| doe |
| woodpecker |
| bunny |
| moose |
| human |
| monkeys |
| ungulates |
| baboon |
| parakeets |
| tapir |
| mutt |
| hunter |
| cetacean |
| raccoons |
| alpaca |
| caterpillar |
| pets |
| lynxes |
| mosquito |
| alligators |
| stoat |
| mammals |
| millipede |
| condor |
| racoon |
| fur |
| rat |
| pup |
| nematode |
| carnivores |
| malamute |
| lemurs |
| hedgehog |
| humane |
| tyrannosaurus |
| kangaroo |
